# A tamper-resistant timed secure data transmission protocol based on smart contract

**DOI:** 10.1038/s41598-023-38136-3

**Published:** 2023-07-17

**Authors:** Ke Yuan, Haowen Cao, Suya Zhang, Chenxu Zhai, Xiaoyu Du, Chunfu Jia

**Affiliations:** 1 School of Computer and Information Engineering, Henan University, Kaifeng, 475004 China; 2 Henan Province Engineering Research Center of Spatial Information Processing, Henan University, Kaifeng, 475004 China; 3 College of Cybersecurity, Nankai University, Tianjin, 300350 China

**Keywords:** Engineering, Electrical and electronic engineering

## Abstract

Many time-sensitive scenarios need to decrypt data at a specified time. The timed-release encryption (TRE) primitive can meet this requirement. However, in the single-time server TRE model, there is a single point of failure problem. Therefore, we propose a tamper-resistant timed secure data transmission protocol based on smart contracts. Firstly, by decomposing the ciphertext into ciphertext fragments, the amount of deposit that a single middleman needs to submit is reduced. Secondly, it provides the system with security redundancy that changes with the decomposition mode. Thirdly, the sender is required to submit the hash value of each ciphertext fragment to the blockchain network at the same time as sending data, so that the receiver can quickly verify the authenticity of the ciphertext to resist substitution attack. Security analysis shows that the proposed protocol model can resist interruption attacks, release-ahead attacks and replacement attacks. Finally, we conduct a monetary cost test on the Ethereum’s Rinkeby test network. The results show that our running cost is almost double compared with the existing similar scheme, but it is still very low and almost negligible compared with the value of the content and the expected profits it brings.

## Introduction

Timed-release encryption (TRE)^1,2^ solves the problem of “sending a message that can only be decrypted after a specified time”. It brings great convenience to many time sensitive scenarios in real life, such as bidding system. The existing TRE schemes are mainly divided into two models: time-lock puzzles (TLP)^1,3–5^ and agents. The TLP schemes implement TRE by solving some non-parallel computing problems. Due to the non-parallelism of the problem design, the time of solving the problem can be estimated which will be taken as the time control of decryption. Agents are divided into interactive server^1,6–9^ and non-interactive server^10–19^ models. In both ways, a “time server” is set up, and the decryption will be accomplished in the future by the time trapdoor released by the time server at a specified time. In the TLP model, the receiver needs to start complex computation immediately after receiving ciphertext, so as to ensure that the decryption can be completed approximately after the designated decryption time arrives. It is difficult to guarantee the precise time while it consumes a lot of computing resources. In the time server model, a precise decryption time can be obtained; however, at present, no arbitrary time decryption control can be provided, and once the time server is attacked or the time server maliciously launches the attack, the security of the model will be seriously challenged.

The secret sharing^20,21^ and blockchain^22–26^ techniques have brought new ideas to solve the above problems for TRE technology. The secret sharing can split a secret into multiple secret shares, allowing partial secret shares to reconstruct the complete secret, and making confidential data have security redundancy characteristics. The blockchain was first formally proposed in 2008 by an anonymous scholar named “Nakamoto”^27^. The public blockchain network is generally regarded as a data ledger maintained by various decentralized nodes. Each node generates a block consisting of blockhead and Merkle tree^28^ through a specific consensus algorithm, such as Proof of Work (PoW)^27^ or Proof of Stake (PoS)^29^. The Merkle tree contains transaction information in the blockchain network for a period of time, including transactions, consensus processes, addresses of newly registered smart contracts^30,31^.

In 2015, Liu et al.^32^ proposed a new timed-release encryption based on Bitcoin protocol and SAT witness encryption. Compared with the TLP schemes, its remarkable advantage is that the decryptor does not need to calculate all the time after receiving the ciphertext, and the decryption key is automatically obtained after the newly generated blocks arrive at a count specified in advance. However, like the TLP schemes, it is difficult to guarantee a precise decryption time. In 2018, Li and Palanisamy^33^ proposed a TRE scheme of data timed transmission (Decentralized Release of Self-emerging Data, DRSD) protocol. In their DRSD protocol, Li et al. used the nodes in the blockchain network as middlemen, and the middlemen were responsible for delivering the decryption key to the receiver after a specified time. The DRSD protocol can provide a precise decryption time and does not require a third-party time server. It is also the most advanced solution for implementing timed-release encryption based on blockchain nodes up to now.

But in the DRSD protocol, there are also some problems. Firstly, the DRSD protocol requires each middleman to provide a deposit in excess of the value of the transmitted content. If the sender wants to transmit a tender for a bidding project, it means that each middleman has to pay a large deposit. This can lead to the high cost of middlemen, and even no middleman can afford the deposit. Secondly, the DRSD protocol has no security redundancy, and any violation of middleman will lead to transmission failure. Finally, although the DRSD protocol uses blockchain technology, it does not use a tamper-resistant modification of blockchain to deal with possible replacement attacks.

In order to solve the above problems, we propose a new tamper-resistant timed secure data (TTSD) transmission protocol. The main contributions of this paper are as follows.

1. We propose a mechanism for tamper-resistant timed data transmission based on smart contracts using blockchain nodes as middlemen. It can be used to solve the protection problem of time sensitive data in artificial intelligence, Internet of Things and other fields.
2. We propose a new deposit mechanism that makes the deposit of each middleman needs to bear decrease proportionally with the increase in the number of middlemen. In addition, the combination of this mechanism with the reporting mechanism enables our protocol to resist interruption attacks and release-ahead attacks and replacement attacks (initiated by some middlemen).
3. We use two ciphertext decomposition methods to obtain data security redundancy, one is our own matrix decomposition method, the other is the existing secret sharing method. When using our matrix ciphertext decomposition algorithm, both decomposition and reassembly require very small resources and can be implemented using simple code. At the same time, we provide 30 to 50% security redundancy for the protocol according to the different ways of ciphertext decomposition.

## Preliminary

In this section, we give a table of key notations for this paper and two ciphertext decomposition methods: our matrix decomposition method and secret sharing method that will be used in our scheme.

### Key notations

For presentation convenience, we list the key notations used in our work in Table 1.

**Table 1:** Key notations used in this paper.

Notation	Interpretation
$\text{ TRE }$	Timed-released encryption
$\text{ DRSD }$	Decentralized release of self-emerging data
$\text{ TTSD }$	Tamper-resistant timed secure data
*S*	The sender who is the initiator of the entire protocol
*Peers*	The middleman cluster which is a collection of peer entities selected by the sender *S*
*R*	The receiver who receives all ciphertext fragments from the middleman cluster at the specified time
$T_s$	The time point when the sender *S* sends the ciphertext fragments to the middlemen
$T_r$	The time point when the middlemen send the ciphertext fragments to the receiver *R*
*c*	The ciphertext
*A*	The matrix
*u*	The size of the minimum ciphertext fragment base
$C_{TTSD}$	The smart contract corresponding to the TTSD protocol
$C_{DRSD}$	The smart contract corresponding to the DRSD protocol
*PubKey*	The public key of the middleman
*PrivKey*	The private key of the middleman
$d^s$	The frozen funds of a middleman
$d^a$	The circulating funds of a middleman
$r_c$	The middleman’s work cost
$\alpha$	The middleman’s actual income
*v*	The value of the content (ciphertext) sent by the sender *S*
*P*	The total revenue obtained in the case of successful sending and winning competition
$Num_{peers}$	The number of middlemen
$b_i$	The bribe provided by the attacker
$b_{peers}$	The total amount of bribery offered by the attackers

### Matrix decomposition method

We propose a matrix decomposition method for ciphertext decomposition shown in Algorithm 1.

**Figure:**
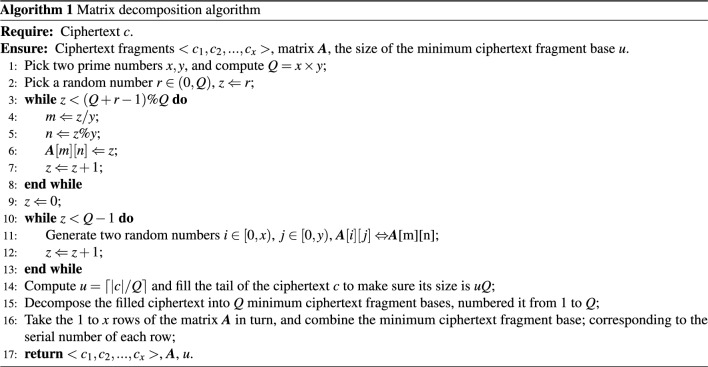

When using the matrix decomposition algorithm, the sender can decompose the ciphertext *c* into *x* ciphertext fragments, and the size of each ciphertext fragment is *uy*. Unless the attacker illegally acquires all the ciphertext fragments, matrix ${{\varvec{A}}}$ and the size of the minimum ciphertext fragment base *u*, the attacker cannot obtain the complete original ciphertext *c*.

The sender can inform the receiver of ${{\varvec{A}}}$ and *u* after the ciphertext decomposition. The receiver can recover the ciphertext after obtaining all the ciphertext fragments. The ciphertext recovery algorithm is shown in Algorithm 2.

**Figure:**
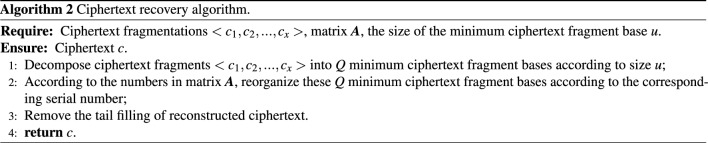

When using the matrix decomposition algorithm, the resources that are consumed by decomposition or reorganization are very small and can be realized with simple code. Regarding the amount of deposit that the middleman needs to submit when using the matrix method, we will discuss it in detail in Sect. Service setup.

### Secret sharing method

TTSD scheme adopts (*t*, *n*) threshold secret sharing method^20^, which refers to the decomposition of a secret *K* into *n* shares, and any *t* or more shares are able to reconstruct the secret *K*. In the TTSD scheme, the sender can use secret sharing scheme to generate *n* ciphertext fragments for transmission.

It should be pointed out that the setting of the value of *t* is the result of the game between the security and reliability of the secret sharing method. If the value of *t* is too low, the security will be reduced, that is, the attacker only needs a small amount of ciphertext fragments to get the complete ciphertext. If the value of *t* is too high, the reliability of the ciphertext will be reduced, that is, the attacker only needs to destroy a small amount of ciphertext fragments to cause the ciphertext cannot be correctly reconstructed.

## Basic scheme

We outline the participants, basic objective, workflow and attack modes of the proposed TTSD protocol in this section. The implementation of the TTSD protocol is maintained and supported by the contract $C_{TTSD}$ in a way similar to Li et al’s scheme.

### Participants

The TTSD protocol involves three participating entities.

${\textbf {Sender}}({\varvec{S}}).$ The sender *S* is the initiator of the entire protocol. *S* calculates and generates ciphertext fragments and selects the required middlemen to form a middleman cluster, and sends tamper-proof content to the blockchain.

${\textbf {Middleman cluster}}({\varvec{Peers}}).$ The middleman cluster *Peers* is a collection of peer entities selected by the sender *S*. They pay a deposit to the contract $C_{TTSD}$ as credit support to receive a task and get the reward.

${\textbf {Receiver}}({\varvec{R}}).$ The receiver *R* receives all ciphertext fragments from the middleman cluster at the specified time $T_R$, and then combines the fragments to obtain the complete ciphertext and decrypts it to generate the plaintext *m*.

### Basic objective

**Figure 1:**
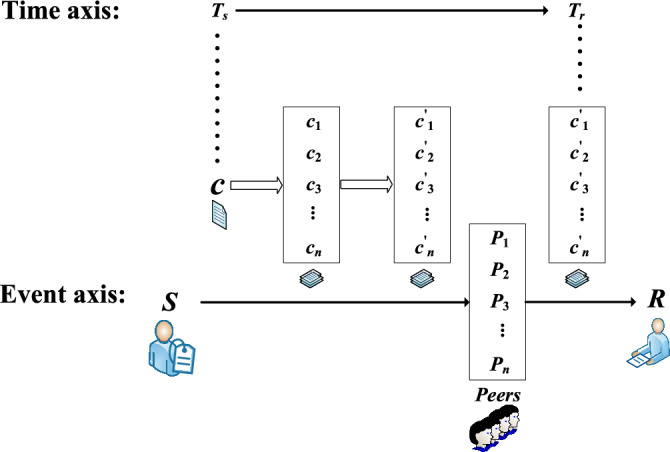
The life cycle of the TTSD protocol.

The basic objective of the TTSD protocol is to achieve timed secure data transmission by middleman cluster, and to ensure that the whole system is in Nash equilibrium. The life cycle of the TTSD protocol is shown in Figure 1.

In order to achieve this objective, we divide the transmitted ciphertext *c* into equal values. We give two kinds of decomposition methods (see Sect. Preliminary for details) and calculate their security thresholds (see Sect. Security threshold for details). At the same time, we need to ensure that the cost is acceptable when the whole TTSD protocol is implemented by the smart contract $C_{TTSD}$. We use Solidity language in the Rinkeby test network of Ethereum for cost testing, which will be explained in detail in Sect. Cost testing and comparison.

In order to better analyze the risks faced when the contract $C_{TTSD}$ is running, we make the following three reasonable assumptions about the actual running scenario of the $C_{TTSD}$:

${\textbf {Limited value.}}$ The value of each ciphertext is limited, which can be approximately equal to the profit of sender *S* after the ciphertext transmission is successful.

${\textbf {Similar strength.}}$ In a project, more than one sender will send files to the same recipient, and these senders have similar competitiveness.

${\textbf {Rational attack.}}$ We assume that the attackers are rational and will not launch attacks regardless of cost.

### Workflow

In this subsection, we give a brief introduction to the four stages of the protocol, explaining the life cycle of the protocol and the work to be done in each stage. This will be explained in detail in Sect. Tamper-resistant timed secure data transmission protocol.

${\textbf {Peer registration.}}$ At any point in time, a new peer can register by paying a security deposit to the contract $C_{TTSD}$ to be added to the registration list maintained by $C_{TTSD}$. The middleman registration module is used to register a blockchain network node as a middleman. The middleman generates his own public-private key pair $<PubKey, PrivKey>$ and declare to the contract $C_{TTSD}$ the total amount of funds he can provide. The protocol requires the middleman to provide a deposit for each service as its credit support.

${\textbf {Service setup.}}$ The sender S needs to select enough middlemen in the service setting phase, calculate the deposit that each middleman needs to pay, and provide remuneration for the middleman. The middleman’s remuneration will not be sent directly to each of the middlemen’s accounts. This part of the funds will be deposited first in the $C_{TTSD}$ contract’s address until recipient R confirms that he has received the complete and correct content.

${\textbf {Service running.}}$ The service runtime stage is the [$T_s$, $T_r$] period. Before time $T_s$, the sender *S* needs to encrypt the ciphertext fragments using the middlemen’s public keys of the corresponding path and send it to the middlemen at time $T_s$. The middlemen need to save the ciphertext fragments in the [$T_s$, $T_r$] time period and make the first decryption to get the original ciphertext fragments when the time $T_r$ is near. At the moment $T_r$, the middleman transmits the ciphertext fragments to the receiver R to complete the service.

${\textbf {Reporting attacks.}}$ In response to a possible attack, the contract $C_{TTSD}$ encourages any node to report the offender to it. Once a middleman’s violation is confirmed, the deposit submitted by the middleman for the service will be confiscated and a portion of the whistleblower used to motivate the violation will be extracted.

### Contractual participants

Under the assumption that the attacker is rational, we consider three possible attacks: interrupted attack, release-ahead attack, and replacement attack. We design the deposit mechanism and reporting mechanism for our contract $C_{TTSD}$ against possible attacks. Detailed coping styles will be explained in Sect. Tamper-resistant timed secure data transmission protocol.

${\textbf {Interrupt attack.}}$ Interrupt attack means that the middleman discards his own saved ciphertext fragment, in an attempt to make the ciphertext not recover properly. Since the middleman has submitted a deposit for each service, the middleman will not launch an attack for no reason. One reason for the middleman to launch an attack is that the middleman has been bribed. Bribery may occur when the briber pays less than the possible gains.

${\textbf {Release-ahead attack.}}$ The early release attack means that the middleman transmits the ciphertext fragment to the receiver R before the specified $T_r$ time. Release-ahead attacks are also accompanied by bribery. But unlike interrupted attacks, the occurrence of release-ahead attacks means that recipient R is also involved in bribery.

${\textbf {Replacement attack.}}$ The replacement attack means that the middleman generates a forged ciphertext fragment using the public key of the receiver R, thereby causing the receiver R to fail to correctly recover the generated plaintext after receiving all the ciphertext fragments. Similar to interruption attacks, substitution attacks can also occur accompanied by the same bribery. But the difference is that the replacement attack uses a fake ciphertext fragment instead of a real one. This kind of attack is more difficult to detect and prove.

## Tamper-resistant timed secure data transmission protocol

In this section, we describe the four stages of the proposed TTSD protocol in detail.

### Peer registration

Peer registration is the first stage of the TTSD protocol. Any peer(node) in the blockchain network can be registered as a candidate middleman in the smart contract $C_{TTSD}$. When a peer registers as a candidate middleman, it needs to pay a deposit to the address of $C_{TTSD}$ as its credit support. Meanwhile, the middleman needs to generate its own public-private key pair $<PubKey, PrivKey>$. The protocol for the registration of any peer is as follows:

**Table:** 

Peer registration protocol	
**a.** Any peer need to pay a deposit and its public key *PubKey* to the $C_{TTSD}$ when registering.	
**b.** Any peer can only use unfrozen funds as new deposits.	
**c.** Any peer can only be cancelled when no funds are available as deposits.	

TTSD protocol requires the peer to provide funds *d* as a deposit source, and in order to enable a peer to perform multiple services simultaneously, the protocol does not require the peer to provide all his own funds for service as a deposit. Therefore, in the course of service, the peer’s funds *d* will be divided into two parts: frozen funds $d^s$ and circulating funds $d^a$, that is $d=d^a+d^s$. The only funds that a peer can freely use are circulating funds, which can be used to pay the deposit for the next service, or can be withdrawn from their accounts at any time by a peer. It should be noted that when the funds are used as deposits, the ownership of the funds will be temporarily transferred to the location of the $C_{TTSD}$ until the normal end of the service. The only way to thaw the deposit and return it to the peer for circulation is to complete the service and the peer has not violated the rules.

When a middleman applies for cancellation, the contract $C_{TTSD}$ checks whether the middleman still has the deposit unfrozen. If not, $C_{TTSD}$ removes the applicant from the list of candidate middlemen and returns all his funds; if there is still a deposit that has not been thawed, $C_{TTSD}$ will reject his application.

### Service setup

Service setup is the second stage of the TTSD protocol. It enables the sender *S* to select suitable middlemen from the list of candidate middlemen at any time to form a middleman cluster. Before service setup, the middleman needs to decompose the ciphertext *c* into several ciphertext fragments $<c_1,c_2,c_3,...,c_n>$. We will adopt two decomposition methods: matrix decomposition and secret sharing.

The protocol for the service setup is as follows:

**Table:** 

Service setup protocol	
**a.** Before time $T_S$, the sender *S* computes the reward *r* required for the contract $C_{TTSD}$ and the deposit $d^s$ submitted by each middleman, and selects the middleman from the list of candidate middlemen.	
**b.** At time $T_S$, the sender *S* sends the ciphertext fragments, the list of the middleman cluster and the middlemen’ reward *r* to the contract $C_{TTSD}$.	
**c.** The contract $C_{TTSD}$ verifies whether the middleman selected by *S* has sufficient circulating funds $d^a$ to pay the deposit $d^s$. If all $d^a>d^s$ are true, the service will start; if any middleman’s $d^a>d^s$ is false, the service will be refused and the sender *S* is asked to re-select the middlemen.	

The sender *S* needs to pay a sum of money *r* for each service, which will be used to pay the middleman’s remuneration. At the same time, because in the Ethereum environment, each invocation of the contract $C_{TTSD}$ will consume a certain amount of resources, the actual payment of the sender includes the middleman’s work cost $r_c$ and actual income $\alpha$.

How to reasonably calculate the deposit that the middleman needs to pay is an important issue that the contract $C_{TTSD}$ needs to solve. Assuming that the value of the content sent by sender *S* is *v*, the total revenue obtained in the case of successful sending and winning the competition is *P*. Suppose that there are multiple senders *S* sending bids to the same receiver *R* in a project, and these senders have similar competitiveness, and there are no sender competitors in the middleman cluster, that is, the middleman does not retaliate against the sender. When competitors (senders) have similar strengths, each participant has the same probability to win. Therefore, each sender’s ciphertext has a value of *kP* where $0<k=\frac{1}{N}<0.5$ when there are *N* competitors. At the same time, no matter which decomposition method is adopted, the ciphertext is decomposed equivalently, so the attacker will pay an equivalent bribe $b_i$ for each middleman who bribes, obviously $b_i>0$. Assuming that the number of middlemen is $Num_{peers}$ and the total amount of bribery offered by attackers is $b_{peers}$. Then $b_{peers}<kP$ is obvious, otherwise the attacker’s cost of bribery will be higher than the proceeds of the bribe. According to $b_{peers}=Num_{peers} \times b_i$, $b_i<\frac{kP}{Num_{peers}}$ can be obtained.

Since the middleman’s acceptance of bribes will inevitably lead to the confiscation of deposits $d^s$ by the contract $C_{TTSD}$. Therefore, the attack will be established only when the bribe $b_i$ provided by the attacker is larger than the deposit $d^s$ provided by the middleman. Then only when $d^s>b_i$, that is, $\sum d^s>\sum b_i=b_{peers}>kP$ is satisfied, the attack will not occur.

When the sender uses the matrix decomposition method to generate ciphertext fragments, the attacker can launch an interrupt attack once it successfully bribes any middleman, that is, the attacker’s attack cost is only $b_i$. One feasible way to solve this problem is to send repeatedly, that is, the sender can select multiple middlemen for each ciphertext fragment to send. At this point, the attacker needs to bribe all the middlemen in one of the fragments to successfully launch the interrupted attack.

Assuming that ciphertext *c* generates *n* ciphertext fragments and sends $j (j \ge 2)$ multiple times, a total of *nj* middlemen are needed. We call those who are responsible for transmitting the same ciphertext fragment a group of middlemen, which is denoted as $Peers_{same}$, and “an attacker taking *m* bribes from *nj* middlemen, and at least one $Peers_{same}$ in *m* middlemen, i.e. interrupt attack can succeed” is denoted as event *A*. Then ${\textbf {Pr}}(A)=1-\frac{C^m_n\times j^m}{C^m_{jn}}$, ${\textbf {E}}(A)={\textbf {Pr}}(A)\times kP$. Obviously, if and only if $m>n(j-1)$, ${\textbf {Pr}}(A)=1, {\textbf {E}}(A)=kp$; if and only if $m<j$, ${\textbf {Pr}}(A)=0, {\textbf {E}}(A)=0$.

If the attacker bribes *m* middlemen and $d^s>\frac{kP}{m}$, the interrupted attack will not occur. At the same time, in order to deal with a release-ahead attack and replacement attack, the deposit $d^s$ needs to meet $nd^s>kP$, ie $d^s>\frac{kP}{n}$. Since we can not predict the exact number of middlemen *m* that an attacker may bribe, we can not quantify *m* to calculate the specific range. Our solution to this problem is to calculate the deposit range which meets the last two attacks, i.e. $d^s>\frac{kP}{n}$, which will lead to a “security threshold” in our scheme. Middleman bribery beyond the “security threshold” result in some security risk to our scheme. We will discuss the “security threshold” in detail in Sect. Security threshold.

In summary, when using the matrix decomposition method, the deposit required to be provied by the middleman needs to meet $d^s>\frac{kP}{n}$, and the total amount of deposit all middlemen required to pay is *jkP*.

When the sender uses secret sharing to generate ciphertext fragments, and assumes that there are *n* ciphertext fragments, the attacker can successfully decrypt the ciphertext if he can obtain *t* ciphertext fragments. If an attacker attempts to launch an interrupt attack or a replacement attack, the attacker needs to bribe at least $n-t+1$ middlemen, at which point $b_i\times (n-t+1)<kP$ can be obtained. Therefore, $d^s>b_i$ can be obtained only when $d^s>\frac{kP}{n-t+1}$ is satisfied. It can be seen from the above formula that the smaller the *t* is, the less the deposit $d^s$ is required for each middleman. But if *t* is too small it will cause the launching of the release-ahead attack to be much easier. In addition, in order to launch a release-ahead attack, the attacker will bribe *t* middlemen, at this point $tb_i<kP$ can be got. Therefore, We also need the middleman deposit $d^s$ to meet $d^s>\frac{kP}{t}$. From the above analysis, we can get $n-t+1=t$, that is $t=\frac{n+1}{2}$. In summary, when the sender adopts the secret sharing method, *n* should be an odd number, and $t=\frac{n+1}{2}$. At this time, the deposit $d^s$ of each middleman should meet $d^s>\frac{kP}{t}$, and the total amount of deposit required for all middlemen is 2*kP*.

### Service running

Service running is the third stage of the TTSD protocol. It sets out the specifications that the middleman should follow during transmission. Any violation will be treated as a violation of the contract $C_{TTSD}$, and once the violation is confirmed, the deposit submitted by the middleman will be confiscated by the $C_{TTSD}$. At the end of each middleman’s decryption operation, a certificate signed by his private key and the hash value of the obtained ciphertext fragments are sent to $C_{TTSD}$ as proof of his compliance with the protocol. The Ethereum’s smart contracts code ensures that the operation of the protocol is enforced. Finally, we analyze the protocol in a behavior tree manner to ensure that the attacker’s attack costs more than the benefit or that any rational middleman will be honest to comply with the protocol.

The protocol for the service running is as follows:

**Table:** 

Service running protocol	
**a.** Before $T_s+|T_t |$, the sender submits the hash value of ciphertext *c* and all the ciphertext fragments $<c_1,c_2,\ldots ,c_n>$ to the contract $C_{TTSD}$, and uses the public key of the corresponding middleman on the path to encrypt ciphertext fragmentation to get $<c'_1,c'_2,\ldots ,c'_n>$.	
**b.** At time $T_s$, the sender sends all ciphertext fragments to the corresponding middleman, and sends the hash values of ciphertext fragments and other public parameters to the receiver *R*.	
**c.** Before $T_r+|T_t |$, the middleman decrypts the ciphertext $c'_i$ transmitted by himself to obtain $c_i$, and automatically sends a certificate signed by his own private key to the $C_{TTSD}$ under the control of the $C_{TTSD}$ code.	
**d.** At time $T_r$, the middleman sends a ciphertext fragment to the receiver, and automatically sends a certificate signed by his own private key and hash value of the ciphertext fragment to the $C_{TTSD}$ under the control of the $C_{TTSD}$ code.	
**e.** The receiver *R* checks whether each middleman sends ciphertext fragments correctly. If correct, the $C_{TTSD}$ refunds the deposit $d^s$. Otherwise, the $C_{TTSD}$ will detect the middleman’s behavior based on the allegations of the recipient *R*, confiscate the deposit of the guilty middleman.	

In response to interrupt and replacement attacks, the contract $C_{TTSD}$ requires the middleman to automatically submit to it a certificate signed by his private key and the hash value of the content that the sender sent. As the $C_{TTSD}$ runs, this information will be stored permanently on the blockchain. Objectively, this evidence cannot be tampered with unless an attacker has more than 50% of the network’s computing power.

The first certificate signed by the middleman indicates the time $T_e$ when the decryption is completed. If the value of $|T_e-T_r|$ is too large, it means that the middleman takes a lot of time in advance to decrypt, at this point, the possibility of the middleman accepting bribes and releasing the ciphertext in advance will increase. The second certificate released by the middleman indicates the time $T_p$ of sending ciphertext fragments to the receiver *R*. If $T_p$ is earlier than $T_r$, it means that the middleman launched a release-ahead attack. Although no matter which way we use to generate ciphertext fragments, the advance transmission behavior of a single middleman will not make the release-ahead attack successful, the middleman’s behavior will still be considered as violation according to the contract $C_{TTSD}$. $C_{TTSD}$ takes part of the middlemen’s violating deposit *a*, as a reward for the behavior reporter, and uses the rest to compensate the sender *S* (multiple middlemen take bribes) or reward other honest middlemen.

Since the public blockchain network currently generates one block every 10 minutes, the recording of time $T_p$ in the blockchain network will have an error of about 10 minutes from the actual situation. But 10 minutes is not enough time for bribers to modify their own documents. In order to deduce the effect of middleman behavior on protocol security, we design a behavior tree for service running protocol to analyze the benefits of different choices of protocol participants, which will be discussed in detail in Sect. Participant behavior tree.

### Reporting attacks

Reporting attacks is the last stage of the TTSD protocol, which is shown as follows:

**Table:** 

Reporting attacks protocol	
**a.** Any registered middleman can submit an attack report to the contract $C_{TTSD}$.	
**b.** The report should contain a certificate signed by the private key of the violating middleman.	
**c.** Once the attack is confirmed, the deposit of the violating middleman will be forfeited. Some of them will be used as a reward *a* for the informer, while others will be used to compensate the sender or to reward honest middlemen.	

Service running protocol requires all middlemen to automatically send a certificate signed by their private key to the contract $C_{TTSD}$ under the control of the $C_{TTSD}$ code while decrypting and transmitting ciphertext. The generation time of these two certificates can be used as a proof of the middleman’s behavior time. Every middleman who tries to report a violation in order to obtain a reward shall submit to $C_{TTSD}$ the information of a private key signature certificate of the middleman being reported. According to the information provided by the informer, the $C_{TTSD}$ can find the certificate in the blockchain network, and the attribution of the certificate can be verified by using the reported person’s public key. If the reported person is found to have violated the rules, his deposit $d^s$ shall be confiscated and a reward shall be given to the informer.

In this reporting mechanism, the best strategy for any rational middleman is to report the violator to the contract $C_{TTSD}$ for reward *a*. Therefore, any rational middleman will not accept bribes less than its deposit $d^s$. At the same time, because each reporting operation will consume a certain amount of Gas as the “fuel” to invoke the $C_{TTSD}$, so any rational middleman will not report the non-violating middleman. In summary, the attack reporting module of $C_{TTSD}$ in this case is in a Nash equilibrium.

## Security and cost analysis

### Security analysis

In this subsection, we conduct security analysis from two aspects: participant behavior tree and security threshold.

#### Participant behavior tree

We design a behavior tree for service running protocol, and analyze the benefits of senders, middleman clusters and bribers under various circumstances to illustrate the security of the scheme. Considering that the proof of matrix decomposition method requires a large number of middlemen, which will lead to the exponential growth of the number of behavior leaf nodes, we take the secret sharing method as an example to illustrate. It should be pointed out that for the matrix decomposition method, the behavior tree is only different in the amount of deposit, and the conclusion is still valid. In Sect. Security threshold, we discuss in detail the security thresholds that may arise in matrix decomposition method. In order to facilitate explanation, we only designed three middleman nodes in the tree, but the situation is the same when there are more middlemen. Although there are only sender *S* and receiver *R* in the middleman list, we assume that the attacker knows the contents of the middleman list in some way, and then bribes the middleman and launches an attack. Every middleman will face the choice of accepting or rejecting bribery. We assume that there is no interaction between the middlemen and the choice of other middlemen can not be known.

**Figure 2:**
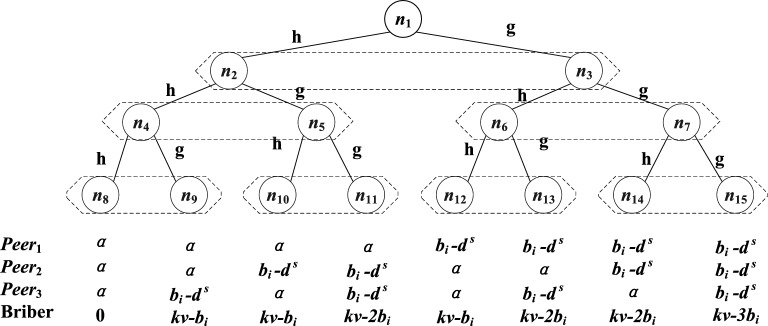
The behavior tree of participant.

The behavior tree of participants is shown in Figure 2. We use *h* to represent the honest behavior of nodes, and *g* to represent the dishonest behavior of nodes. In this figure, the honest behavior of middleman $Peer_1$ in the $n_1$ node gives birth to the $n_2$ node, and dishonest behavior gives birth to the $n_3$ node. Similarly, $Peer_2$ gives birth to $n_4\sim n_7$ nodes; $Peer_3$ gives birth to $n_8\sim n_{15}$ nodes. According to the deposit calculation formula in Sect. Service setup, the deposit $d^s$ in this figure should satisfy $d^s>0.5kP$, while the bribe $b_i$ meets $b_i<0.33kP$.

As can be seen from Figure 2, in the case of $n_{15}$, the cost of bribery by the attacker is greater than the benefit, so the attacker will not launch an attack. In the case of $n_9$, $n_{10}$, and $n_{12}$, the attack cannot succeed, so the attacker will not launch the attack. The result of $n_{11}$ is that $peer_2$ and $peer_3$ accept bribes. At this time, the income of the middleman is $b_i-d^s$. It can be seen from $d^s>0.5kP>b_i$ that the bribery received by the middleman can not compensate for the loss of its deposit, so $n_{12}$ will not occur. Similarly, $n_{13}$ and $n_{14}$ will not occur, and the best strategy for middlemen is to abide by the contract $C_{TTSD}$ under any circumstances.

#### Security threshold

In this subsubsection, we quantitatively analyze the security thresholds of the system when using secret sharing method and matrix decomposition method.

*A. Secret sharing method*

In Sect. Service setup, we draw a conclusion that when using secret sharing as ciphertext decomposition method, the sender *S* should make $t=\frac{n+1}{2}$ (*n* is odd) in order to deal with interrupt attack, replacement attack and early release attack. Therefore, only when the attacker destroys or forges more than half of the ciphertext fragments can the interrupted attack or replacement attack occur. If an attacker tries to launch a release-ahead attack to decrypt it in advance, it also needs to bribe more than half of the middlemen to launch the attack. Therefore, the security threshold of the secret sharing scheme is 50%. But at the same time, because the system requires total deposit $\Sigma d^s>2kP$ of all the middlemen, objectively, any attack is worthless if the attacker is rational.

*B. Ciphertext decomposition method*

The matrix decomposition method adopts the method of *j*-times repetitive transmission of *n* ciphertexts to prevent attacks. This method looks primitive and vulnerable, but according to our test, it can still make an attacker’s attack somewhat more difficult.

**Figure 3:**
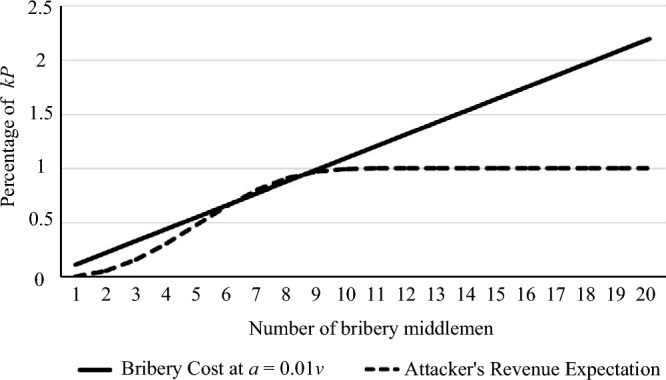
Contrast between bribery cost and mathematical expectation of bribery.

When an attacker tries to bribe a middleman, the briber needs bribe $b_i$ to satisfy $b_i>d^s+\alpha$. Otherwise, when the middleman accepts bribes, the profit will be less than the profit when the middleman chooses to abide by the $C_{TTSD}$. Accordingly, we can carry out the quantitative analysis. We assume that there are 10 ciphertext fragments and each ciphertext fragment is sent twice. The relationship between the cost of the attacker launching an interrupt attack or replacement attack and the mathematical expectation of bribery success is shown in Figure 3.

As can be seen from Figure 3, when more than nine of the twenty middlemen are bribed, the bribery $\Sigma b_i$ of the briber approaches *kP*. When the attacker bribed 6 middlemen, the mathematical expectation of successful bribery would be close to the cost of bribery; when the attacker bribed more than 7 middlemen, the mathematical expectation of a successful attack would exceed the cost of attack. Therefore, the security threshold of matrix decomposition method should be 30%. It should be pointed out that even if the attacker bribes 7 middlemen to successfully launch an interrupt attack or replacement attack, the attacker’s income is only 0.23*kP*, which means that nearly 80% of the attacker’s project income will be used to pay briberies.

At the same time, in the above scenario, the attacker needs to bribe at least half of the middlemen to launch a release-ahead attack, and the bribery cost will be greater than the benefits, so the release-ahead attack will not happen.

### Cost testing and comparison

We use a regular personal computer with an Intel Core i5-6300HQ processor to test in the Ethereum *Rinkeby* test network using *Solidity* language. This test is to verify the approximate amount of Gas consumed in each step in the Ethereum environment. Gas is the “fuel” of Ethereum network smart contracts. Every registration and execution of smart contracts requires a certain amount of Gas. The exchange ratio between Gas and ETH currency is about 1 ETH = $$1\times 10^9$$ Gas. When we conducted the experiment on June 19, 2022, the exchange ratio between USD and ETH currency 1 EHT = $938.34^34^. Thus, the number of Gas consumed by each function in the smart contract $$C_{TTSD}$$ and its converted ETH and USD price were shown in Table 2.

**Table 2:** The cost of function running in the smart contract $C_{TTSD}$.

Stages	Functions	Gas cost	USD cost	ETH cost
Peer registration	newPeers	278774	$0.26	0.00028
	updataBalance	33476	$0.03	0.00003
Service setup	senderSign	245692	$0.23	0.00025
	recverSign	21661	$0.02	0.00002
	setUp	1247561	$1.17	0.00125
Service running	peerToRecver	121705	$0.11	0.00012
	setCert	21345	$0.02	0.00002
Reporting attacks	recove	72423	$0.07	0.00007
	report	104271	$0.10	0.00010
	award	72743	$0.07	0.00007

The *newPeer*() function in Table 2 is used when nodes in the Ethereum network try to register as middlemen, which is not consumed within a service. *updataBalance*() is used to update the deposit information of the middleman. *senderSign*() and *recverSign*() are used to confirm the information of the sender and receiver. When the sender *S* needs to start the service, the *setUp*() is used to provide relevant information to the contract $C_{TTSD}$. In each service process, each middleman needs to call *peerToRecver*() once and *setCert*() twice. When a middleman tries to report a violator, it uses the *report*() to send a report to $C_{TTSD}$. The report uses the *recove*() function to verify whether the certificate reported by the whistleblower is true, and ultimately uses the *award*() to determine whether the reported person is in violation. This portion of Gas consumption will be temporarily paid by the whistleblower and offset by the reporting reward after the report is successful. To sum up, in the case of Sect. Ciphertext decomposition method, the cost of Gas consumption for a single transmission is about $setUp()+20peerToRecver()+40setCert()=\$4.67$.

We compare the running costs of $C_{TTSD}$ and $C_{DRSD}$ contracts. We assume that $C_{DRSD}$ requires three middlemen (as was the case in the experiments of DRSD), while our $C_{TTSD}$ requires 20 middlemen. Assuming two repeats, the middlemen will have to pay a total deposit of 2*kP*, regardless of the decomposition mentioned in the article. Meanwhile, we compare $C_{TTSD}$ and $C_{DRSD}$ contracts in terms of the number of content transfers (denoted as *Send*) and the number of signature certificates sent by middlemen (denoted as *Cert*). The results are shown in Table 3.

**Table 3:** Cost comparison between TTSD and DRSD.

Protocol	Cost	Sender	Peer	Deposit
TTSD	$4.67	20 Send	20 Send+40 Cert	0.1*kP*
DRSD	$2.38	1 Send	3 Send+3 Cert	*kP*

Due to a large number of middlemen, the use cost of $C_{TTSD}$ has a certain increase compared with $C_{DRSD}$, but $C_{TTSD}$ can reduce the deposit required by each middleman to one-tenth of $C_{DRSD}$, which will greatly reduce the financial pressure of middlemen and increase the number of available middlemen. Obviously, with the increase of the number of middlemen in a single service of $C_{TTSD}$, its cost will slightly increase, but its security threshold will also increase, and the deposit to be paid by a single middleman will also be proportionally reduced. At the same time, because $C_{TTSD}$ is mainly used for services which transmit content of relatively high value, the extra cost can be almost ignored. According to the different ways of ciphertext decomposition, the security threshold of $C_{TTSD}$ is about 30% or 50%, it provides higher security redundancy compared with $C_{DRSD}$, which provides higher reliability and security for the contract.

## Conclusion

In this paper, we propose a TTSD protocol implemented as a smart contract in the Ethereum blockchain. Compared with the existing DRSD protocol, our TTSD protocol reduces the amount of deposit that a single middleman needs to pay by decomposing ciphertext. For the ciphertext of high value, it increases the number of available middlemen. With the addition of ciphertext fragments, attackers can no longer successfully attack the system only by attacking a single middleman. This provides security redundancy for the system, which will change according to the different ways of ciphertext decomposition. At the same time, the hash value of the sender’s each ciphertext fragment in our protocol will be recorded in the blockchain network, which enables the receiver to quickly verify the authenticity of the ciphertext and prevent replacement attacks. In the future, we will try to design a more secure ciphertext decomposition method which is harder to crack to improve the security of the protocol. We will also consider introducing a reputation mechanism instead of part of the deposit, so as to reduce more financial pressure on the middleman. In addition, we will focus on other specific scenarios, such as cross-domain exchange of IoT data^35^, design special timed transport protocol.

## Data Availability

The datasets used and analysed during the current study available from the corresponding author on reasonable request.
